# Supplementary material for: The uncertainty related to the inexactitude of prioritization based on consistent pairwise comparisons
Source: PLoS One. 2023 Sep 8;18(9):e0290751. doi: 10.1371/journal.pone.0290751 (PMC10490963; doi:10.1371/journal.pone.0290751)
Supplement: Attachment — Submitted filename: ResponseToReviewers.docx [file pone.0290751.s001.docx]

Pawel Tadeusz KAZIBUDZKI

Opole University of Technology,

Faculty of Economics and Management,

St. Luboszycka 7, 45-036 Opole, Poland;

ORCID: 0000-0003-1203-4235

[p.kazibudzki@po.edu.pl](mailto:p.kazibudzki@po.edu.pl)

June 22, 2023

### Responses to the Reviewers

Dear Colleagues – the order of my answers for your reviews was decided to be randomly selected in relation to the editorial system of PLoS ONE and the email we received from the Editorial Office. As I am unaware of your identities thus I will call you Reviewer No. 1 and Reviewer No. 2. The numbers standing next to your anonymous identity are assigned for the order of your appearance in the editorial system of the Journal and my responses identification and have no other meaning whatsoever.

**Reviewer No. 2**

Dear Professor – I would like to thank you for your time and appreciation of my research efforts. I owe you so much for your exceptionally positive judgment of my manuscript. Obviously, I fully fulfilled your suggestion to add the Section “Systematic Review of Literature” in the article and add all data underlying the findings in the Supporting Information Files Section i.e. Pawel Tadeusz Kazibudzki. (2023). Supporting Information Files [Data set]. Zenodo. <https://doi.org/10.5281/zenodo.8071858>. Thanks a lot for your benevolence!

**Reviewer No. 1**

Dear Professor – I would like to thank you for your time and appreciation of my research efforts. I truly appreciate your very positive judgment of the paper. Certainly, all your suggestions to improve my manuscript were applied to its structure and merit. I am going to elaborate details of the manuscript’s improvements in the text hereafter.

Add some main finding in the abstract and also add one or two lines about significance of present study which show why you choose this topic.

– it’s done, please note the following phrases: “This research study is a mile stone regarding the interpretation and reliability evaluation of results for the prioritization process with the application of the pairwise comparisons technique. Firstly, the research study results change the perspective of the rank reversal phenomenon, which shed new light on many research studies that have been presented in the subject’s literature for many decades. Secondly, the research study results throw new light on the discussion concerning the fuzziness of AHP’s results. As known, the creator of the AHP was strictly against further fuzzifying of AHP’s results. Last but not least, the effect of the research opens the unique opportunity to evaluate the prioritization outcome obtained within the process of consistent pairwise comparisons from the well-known perspective of statistical hypothesis testing i.e. the probability evaluation of accepting ranking results which are wrong, and rejecting ranking results which may be right. The paramount finding of the research is the fact that consistent PCMs provide PVs, which elements cannot be considered as established, but only approximated within certain confidence intervals estimated with a certain level of probability.”;

The title is not uniform because some words are start capital and some capital and small letter both.

– the title is uniform at the present moment, it was corrected to the journal requirements.

The introduction. The authors should make it clear why this study is novel.

– it’s indicated now, please note the following phrases: “The issue of in/consistency in PCs is also especially attractive when examined from the perspective of its relation to trustworthiness of Priority Ratios (PRs) derived from PCMs with application of various Priorities Deriving Methods (PDMs). It is believed for example, although some evidence from a few research papers contradicts this belief, see e.g. [76,77,111,112], that inconsistent PCMs provide less credible PRs and inconsistency reduction is the process that leads to betterment of priorities estimation. It is also believed that consistent PCMs and inconsistent PCMs provide Priority Vectors (PVs), denoted as ***w****=*[*w*_1_,…, *w_n_*]^T^, where *w_i_>*0, *i=*1,…, *n*, whose elements are considered as established, and not approximated within a certain confidence interval estimated with a certain level of probability. The latter belief is of special interest of this research and its novelty as it has serious repercussions in relation to decision making quality with the application of PCs methodology i.e. the uncertainty related to the inexactitude of prioritization based on consistent pairwise comparisons. So far, this concept has been studied only from the perspective of inconsistent PCMs, see e.g. [51,76,77,111–114], hence there is a research gap which this research paper intends to fill in.”;

In the Introduction, the literature review was not logically organized and all literatures cited seem separate descriptions without connections. The readers can't know what the state-of-art methodologies or gaps the current study plans to resolve or fill, and how significant or what contribution the current study is?. Rewrite the last paragraph of introduction section and make it concise which contains the objective, novelty, motivation, and why you choose this problem.

– the Introduction section is entirely rewritten now, the questionable lines were removed and relocated to the new section called “Systematic Review of Literature”;

Must add future direction of your research.

– added in the Conclusions Section, i.e. “Future direction of the research may challenge these limitations as well include other preference scales which were not examined in this study.”;

Consequently, it is important to call your attention to the fact that all the sections must be logically connected. Based on the fact that the outcome of a research, after published, is cited and built-upon in the world, it is very important to improve the quality of presentation. Consequently, you need to:

(a) connect the title to the abstract
(b) connect the abstract to the introduction
(c) prepare the introduction to accurately lead to the analysis of results.
(d) divide the discussion of results into (i) analysis of results and (ii) discussion of results.
(e) conclude the report based on the facts you have analyzed and discussed.

Comment: Do the needful. Follow the pattern above to connect all the sections.

– the manuscript was restructured as suggested. I did what was necessary. The sections were updated in accordance with your suggestions. Thank you.

Also the authors are advised to update the manuscript before final submission as it contains some typos and grammatical errors.

– the manuscript was entirely proofread by a professional native speaker, hence it is updated now in accordance with your proposition.

Respectfully yours,

Pawel Tadeusz Kazibudzki, PhD
