# Supplementary material for: The uncertainty related to the inexactitude of prioritization based on consistent pairwise comparisons
Source: PLoS One. 2023 Sep 8;18(9):e0290751. doi: 10.1371/journal.pone.0290751 (PMC10490963; doi:10.1371/journal.pone.0290751)
Supplement: Attachment — Submitted filename: ResponseToReviewers.docx [file pone.0290751.s004.docx]

Pawel Tadeusz KAZIBUDZKI

Opole University of Technology,

Faculty of Economics and Management,

St. Luboszycka 7, 45-036 Opole, Poland;

ORCID: 0000-0003-1203-4235

[p.kazibudzki@po.edu.pl](mailto:p.kazibudzki@po.edu.pl)

August 9^th^, 2023

### Responses to Reviewers and the Academic Editor

The content of this document will be devoted exclusively to suggestions made by the editor as the manuscript at its current stage is fully and unconditionally accepted by two independent reviewers. They both claim that the manuscript is suitable for publication as it stands –

Reviewer #1: All comments have been addressed; The authors revised the manuscript according to the wise suggestion so the revised manuscript is suitable for publication.

Reviewer #2: All comments have been addressed.

The author appreciates comments of the editor perfecting the manuscript and humbly introduce the editor’s suggestions everywhere they lead to the betterment of the research paper.

**Additional Editor Comments**:

I appreciate the effort made by the author and consider some comments in revision. So, please incorporate following rest of the comments.

1. The title of study may be like that ‘The uncertainty in decision making process related to the inexactitude of prioritization based on consistent pairwise comparisons”, “The uncertainty related to the inexactitude of prioritization based on consistent pairwise comparisons”
   The title start with the word “on” may not making a good sense. It is not compulsory for you to choose the propose one. You can also propose as you think suitable.

The author appreciates the editor’s suggestion. The title was positively evaluated by the professional proofreader who is also the American native speaker. American English is not the author’s first language. The author depends on opinions of professionals. Hence, to acknowledge the editor’s viewpoint and appreciate the editor’s input to the problem, the author decided to exchange the word “on” on the word “The”.

2. What is new in your study?” or statements like “Monte Carlo Simulation is an old fashioned approach/tool. In answer you can discuss your contributions even though you are using Monte Carlo Simulation but your answer is not showing good professionalism. Dear Author, thanks for sharing the Journal policy link

(<https://journals.plos.org/plosone/s/editorial-and-peer-review-process>) now I will request you please read the policy very carefully especially the headings “Editor Decision (The editor considers reviewer feedback and their own evaluation of the manuscript in order to reach a decision)”.

The author would like to request the editor to understand that the written language connotations can sometimes be very confusing and sometimes it is very hard to understand the character of the writer’s intentions, especially when the stake is high and professionalism is interwoven with emotions like in this case. The author is fully aware that the “last” word in the case “to publish” or “not to publish” belongs to the editor and the editor’s professional judgment. However, the author would very much appreciate if the editor could choose to notice that the methodological contribution to the state of knowledge related to Monte Carlo Simulations was already indicated in the manuscript in its first and second revisions, please see e.g.

“As it seems the third issue is probably the most important problem in the contemporary arena of the MADM theory concerning AHP, and the only way to examine that phenomena is through computer simulations. It is the fact that Monte Carlo simulations are commonly recognized and applied as important and credible source of scientific information [1,2]. Their applications spread for examination purposes of various phenomena, e.g.: consequences of decisions made, or different processes subdued to random impact of the particular environment [3–10].”,

and/or for example:

“However, only few research studies examine this problem from the perspective of PCM applicability for credible designation of decision maker’s (DM) priorities in the way that leads to minimization of the prioritization uncertainty related to possible, and sometimes very probable, ranking fluctuations. This problem constitutes the primary area of interest for this research paper as no research study was thus far identified that examines this problem via complex simulations from the perspective of consistent PCMs. So far, this concept has been studied only from the perspective of inconsistent PCMs, see e.g. [5,7,11–15]. As problems related to heuristics can be analyzed only via a computer simulation process, because they cannot be mathematically determined, the problem examined in this research paper is examined via Monte Carlo simulations, appropriately coded and executed with the application of Wolfram’s Mathematica Software.

It is the fact that Monte Carlo simulations are commonly recognized and applied as important and credible source of scientific information [1,2]. Their applications spread for examination purposes of various phenomena, e.g.: consequences of decisions made, or different processes subdued to random impact of the particular environment [3–10]. Hence, for the analysis of the problem revealed in the former subsections, the following Monte Carlo simulation algorithm is proposed (Fig 1-2). The primary version of the algorithm was devised and successfully applied for the first time by Grzybowski [14], and since then it was adapted and successfully implemented for many similar problems, see e.g. [5,7,11–13,15–18]. This research study applies the adaptation of this algorithm for examination of consistent PCMs.”.

…, and the fact that the editor indicated that the Monte Carlo Simulation methodology is not new but even an old fashioned concept, was understood by the author as the editor’s claim that the research paper lacks of its novelty to meet the journal’s standards – that is the only reason that these standards were brought to the editor’s attention. The author believes that now the editor will fully understand and appreciate the author’s intentions as fully professional.

3. Review of literature is not relevant to the study. Authors must add relevant studies of fuzzy AHP and traditional AHP method as well as Monte Carlo Simulation algorithm. For example, line No. 188, “the Weighted Product Model (WPM), see e.g. [120, 121]” but I did’t find the name of this method (WPM) in mentioned reference 120.
   Line No. 186: problems: The Weighted Sum Model (WSM), see e.g. [3,118,119], but I did’t find the name of this method (WSM) in mentioned reference 03, Please recheck and answer this point.
   Furthermore, I agreed with author the present moment contains 232 references, so author may reduce this number by deleting old references and focus on more relevant and updated references.

All the relevant studies of fuzzy AHP and traditional AHP method as well as Monte Carlo Simulation algorithm were already added to the manuscript during its first and second revisions. The editor’s suggestion to eliminate indicated references i.e. – Roy B. Decision-aid and decision-making. Eur J Oper Res. 1990; 45: 324–331. doi:10.1016/0377-2217(90)90196-I, and – Velasquez M, Hester P. An analysis of multi-criteria decision making methods. Int J Oper Res. 2013;10: 56–66 – from the indicated positions, was fully applied. However, the author would like his manuscript to be fully supported by related and ample references. Hence, the author would like to kindly request the editor to appreciate the present value of the manuscript’s ample literature as it also may conduce to its readability and number of its quotations.

4. I appreciate the author for proofreading the paper but still there are some sentences that need to revise carefully. Line No, 104-107: Taking into account the AHP drawbacks, many indicators of PCM consistency, commonly known as consistency indices (CIs), have been also proposed thus far, see e.g. [23,24,41,51–68] or, for example [69–77]. They also, due to brevity of this article, will not be scrutinized herein. The sentences should be in a follow. Here are some examples. Please read carefully and improve the write up.

The author appreciates the editor’s suggestion. The questionable sentence was rephrased. American English is not the author’s first language. The author depends on opinions of professionals. The author can only hope that the editor will choose to appreciate the author’s efforts to meet the editor’s requirements.

The author deeply appreciates suggestions of the editor and believes that all improvements introduced to the manuscript as suggested by the editor will be also appreciated by the editor and will meet the editor’s expectations what will entail the unconditional acceptance of the manuscript for publication at its current stage in the PLoS ONE journal.

The author can only believe that the editor will choose to take into account also the fact that the manuscript is already unconditionally approved for publication in the journal by two independent reviewers.

Respectfully yours,

Pawel Tadeusz Kazibudzki, PhD

**References**

1.	Carmone, F.J.; Kara, A.; Zanakis, S.H. A Monte Carlo Investigation of Incomplete Pairwise Comparison Matrices in AHP. *Eur. J. Oper. Res.* **1997**, *102*, 538–553, doi:10.1016/S0377-2217(96)00250-0.

2.	Herman, M.W.; Koczkodaj, W.W. A Monte Carlo Study of Pairwise Comparison. *Inf. Process. Lett.* *57*, 25–29.

3.	Kazibudzki, P.T. The Quality of Ranking during Simulated Pairwise Judgments for Examined Approximation Procedures. *Model. Simul. Eng.* **2019**, *2019*, e1683143, doi:10.1155/2019/1683143.

4.	Zahedi, F. A Simulation Study of Estimation Methods in the Analytic Hierarchy Process. *Socioecon. Plann. Sci.* **1986**, *20*, 347–354, doi:10.1016/0038-0121(86)90046-7.

5.	Kazibudzki, P.T. An Examination of Ranking Quality for Simulated Pairwise Judgments in Relation to Performance of the Selected Consistency Measure. *Adv. Oper. Res.* **2019**, *2019*, e3574263, doi:10.1155/2019/3574263.

6.	Kazibudzki, P.T. An Examination of Performance Relations among Selected Consistency Measures for Simulated Pairwise Judgments. *Ann. Oper. Res.* **2016**, *244*, 525–544, doi:10.1007/s10479-016-2131-6.

7.	Grzybowski, A.Z.; Starczewski, T. Simulation Analysis of Prioritization Errors in the AHP and Their Relationship with an Adopted Judgement Scale. In Proceedings of the Proceedings; San Francisco, USA, October 23 2018; p. 5.

8.	Wu, H.; Leung, S.-O. Can Likert Scales Be Treated as Interval Scales?—A Simulation Study. *J. Soc. Serv. Res.* **2017**, *43*, 527–532, doi:10.1080/01488376.2017.1329775.

9.	Zanakis, S.H.; Solomon, A.; Wishart, N.; Dublish, S. Multi-Attribute Decision Making: A Simulation Comparison of Select Methods. *Eur. J. Oper. Res.* **1998**, *107*, 507–529, doi:10.1016/S0377-2217(97)00147-1.

10.	Winsberg, E. *Science in the Age of Computer Simulation*; University of Chicago Press: Chicago, IL, 2010; ISBN 978-0-226-90204-3.

11.	Kazibudzki, P.T. On Estimation of Priority Vectors Derived from Inconsistent Pairwise Comparison Matrices. *J. Appl. Math. Comput. Mech.* **2022**, *21*, 52–59, doi:10.17512/jamcm.2022.4.05.

12.	Kazibudzki, P.T.; Křupka, J. Pairwise Judgments Consistency Impact on Quality of Multi-Criteria Group Decision-Making with AHP. *EM Ekon. Manag.* **2019**, *22*, 195–212, doi:10.15240/tul/001/2019-4-013.

13.	Kazibudzki, P.T. Redefinition of Triad’s Inconsistency and Its Impact on the Consistency Measurement of Pairwise Comparison Matrix. *J. Appl. Math. Comput. Mech.* **2016**, *15*, 71–78, doi:10.17512/jamcm.2016.1.07.

14.	Grzybowski, A.Z. New Results on Inconsistency Indices and Their Relationship with the Quality of Priority Vector Estimation. *Expert Syst. Appl.* **2016**, *43*, 197–212, doi:10.1016/j.eswa.2015.08.049.

15.	Grzybowski, A.Z.; Starczewski, T. New Look at the Inconsistency Analysis in the Pairwise-Comparisons-Based Prioritization Problems. *Expert Syst. Appl.* **2020**, 113549, doi:10.1016/j.eswa.2020.113549.

16.	Kazibudzki, P.T. The AHP Phenomenon of Rank Reversal Demystified.; December 2022.

17.	Grzybowski, A.Z.; Starczewski, T. Remarks about Inconsistency Analysis in the Pairwise Comparison Technique. In Proceedings of the 2017 IEEE 14th International Scientific Conference on Informatics; November 2017; pp. 227–231.

18.	Grzybowski, A.Z. On Some Recent Advancements within the Pairwise Comparison Methodology. In Proceedings of the 2017 IEEE 14th International Scientific Conference on Informatics; November 2017; pp. 1–5.
